# Editorial: Monocyte Heterogeneity and Function

**DOI:** 10.3389/fimmu.2020.626725

**Published:** 2021-01-07

**Authors:** Florent Ginhoux, Alexander Mildner, Emmanuel L. Gautier, Andreas Schlitzer, Claudia Jakubzick, Chen Varol, Calum Bain, Pierre Guermonprez

**Affiliations:** ^1^ Singapore Immunology Network (SIgN), A*STAR, Singapore, Singapore; ^2^ Translational Immunology Institute, Singhealth/Duke-NUS Academic Medical Centre, the Academia, Singapore, Singapore; ^3^ Shanghai Institute of Immunology, Shanghai Jiao Tong University School of Medicine, Shanghai, China; ^4^ Max-Delbrück-Center for Molecular Medicine (MDC), Berlin, Germany; ^5^ Sorbonne Université, INSERM UMR-S 1166, Hôpital de la Pitié-Salpêtrière, Paris, France; ^6^ Quantitative Systems Biology, Life and Medical Sciences Institute, University of Bonn, Bonn, Germany; ^7^ Single Cell Genomics and Epigenomics Unit at the German Center for Neurodegenerative Diseases and the University of Bonn, Bonn, Germany; ^8^ Department of Microbiology and Immunology, Dartmouth Geisel School of Medicine, Hanover, NH, United States; ^9^ The Research Center For Digestive Tract and Liver Diseases, Sourasky Medical Center and Department of Clinical Microbiology and Immunology, Sackler Faculty of Medicine, Tel Aviv University, Tel Aviv, Israel; ^10^ The University of Edinburgh Centre for Inflammation Research, Queen’s Medical Research Institute, Edinburgh BioQuarter, Edinburgh, United Kingdom; ^11^ King’s College London Centre for Inflammation Biology and Cancer Immunology, The Peter Gorer Department of Immmunobiology, London, United Kingdom; ^12^ Université de Paris, Centre for Inflammation Research, CNRS ERL8252, INSERM1149, Hopital Bichat, Paris, France

**Keywords:** monocytes, macrophages, inflammation, inflammation resolution, cancer, chronic inflammatory disease, cardiovascular disease, fibrosis

Monocytes originate from bone marrow hematopoietic stem cells and circulate in the bloodstream. Monocyte extravasation and differentiation serve multiple immune functions. The differentiation of monocytes into tissue macrophages at steady state can serve homeostatic functions. Monocytes can also fuel acute inflammatory reactions and anti-microbial immunity by differentiating into inflammatory macrophages. Finally, monocytes also actively contribute to the resolution of inflammation and tissue regeneration.

The subset classification of monocytes is a rapidly emerging field. Recent progress in single-cell genomics and high dimensional approaches in phenotyping have highlighted additional subsets of monocytes. Monocytes might also adopt new dynamic transcriptional states associated with inflammation and reflecting their subset heterogeneity. This brings the research community to face a significant challenge of assigning monocyte heterogeneity to specific functions. The goal of this Research Topic is to gather contributions bringing new insights into:

- Monocyte subsets and their ontogeny
- Monocyte heterogeneity and inflammatory diseases
- Monocyte heterogeneity and the regulation of inflammation
- Monocyte heterogeneity and cancer

## Monocyte Subsets And Their Ontogeny

Kapellos et al. provide an updated classification of human monocytes by compiling multiple profiling studies 1. Monocytes are composed of two main subsets: CD14^+^ and CD16^+^ monocytes in humans and Ly6C^high^ and Ly6C^low^ monocytes in mice.

While originally referred to as “inflammatory” monocytes, Ly6C^high^ and CD14^+^ monocytes are now termed “classical” monocytes. Although classical monocytes constitutively enter tissues during homeostasis, upon inflammation, they rapidly extravasate and, depending on the needs of the environment, can differentiate into multiple cell types such as monocyte-derived macrophages.

CD16^+^ and Ly6C^low^ monocytes are termed as “non-classical” (Kapellos et al.). Non-classical monocytes display an intra-vascular function, interacting dynamically with endothelial cells (1).* *In humans, the existence of “intermediate” CD14^+^CD16^+^ monocytes complicates the distinction of “classical” vs. “non-classical” (Kapellos et al.). Expression of 6-sulfo LacNac sugar antigen linked to the cell surface protein PSGL-1 is termed as SLAN. SLAN expression defines non-classical CD16^+^ in humans and provides a practical way to distinguish them from the “intermediate” CD14^+^CD16^+^ monocytes (Hofer et al.).

The developmental pathways by which monocytes arise from hematopoietic stem cells have received a lot of attention. Since the identification and characterization of Granulocyte-Monocyte Progenitors by Akashi et al. in 2000 (2), the ontogenetic pathways of monocytes have been continuously revisited. Geissmann et al. identified progenitors endowed with a mixed potential for monocytes and dendritic cells but devoid of granulocyte potential (3).

In this topic, Wolf et al. review the current line of evidence supporting the existence of multiple differentiation pathways for monocytes (Wolf et al.). GMP-dependent monocytes would co-exist with MDP-derived monocytes. The later encompass monocytes progenitors endowed with a potential for the generation of monocyte-derived DCs (moDCs). Fate mapping studies with *Ms4a3* ^cre^/*Ms4a3* ^CreERT2^ driver lines identifies the GMP-dependent pathway for the generation of monocytes and assesses precisely its contribution to the tissue resident macrophages (TRMs) pool (4).

The differentiation of monocytes into cellular products endowed with DC-like features has been historically evidenced *in vitro* (5). Since then, multiple lines of evidence suggest the physiological relevance of this process. In this topic, Coillard et al. review the current evidence suggesting that moDCs actually accumulate in patho-physiological conditions with important outcomes for the exacerbation or regulation of inflammation (Coillard and Segura).

Duroux-Richard et al. summarize the current of knowledge on the regulation of monocytes subsets by microRNAs. For instance, mir146a regulate the pool of classical monocytes with a regulatory role in osteoclastogenesis (Duroux-Richard et al.).

Assessing developmental pathways underlying the production of monocyte subsets during homeostasis should not obliterate the fact that monocytes are highly dynamic and regulated by both environmental and genetic factors (Kapellos et al.; Patel and Yona). In this topic, Patel et al. and Kapellos et al. provide a fascinating inventory of factors impacting on monocyte populations thereby emphasizing that “steady state is not a single physiological condition” (Patel and Yona) and providing some insights on how changes in lifestyle impact on monocyte subsets (Kapellos et al.).

Deciphering the aegis of specific signals is often complicated by the fact that they act not only on monocytes but also on numerous other myeloid lineages. The inflammatory factor GM-CSF provides a good example of such pleiotropy. Zhan et al. delineate the complex effects on GM-CSF on myeloid lineages and highlight how signal dosage impacts on signaling outcomes and biological functions. Specifically, low doses of GM-CSF activate preferentially the PI3K/Akt while higher doses of GM-CSF are needed to activate the JAK2/STAT5 pathway (Zhan et al.).

## Monocyte Heterogeneity and Inflammatory Diseases

Monocyte subsets represent both circulating precursors and effector populations in the bloodstream or within tissues (Kapellos et al.) (6). For instance, Ly6C^low^ and CD16^+^ monocytes mainly display an intra-vascular function, interacting dynamically with endothelial cells (Hofer et al.) (1). Kapellos et al. extensively review evidences highlighting the modification of the compartment of monocyte associated to COPD, or atherosclerosis for instance.

Monocyte extravasation supports their function of precursors for macrophages. In particular, Dick et al. provide an integrative picture of the remodeling of cardiac macrophages populations imposed by ischemic injury. Some striking features emerging from multiple scRNAseq studies might apply to various cases of acute injury: i) TIMD4^+^LYVE1^+^MHCII^low^ TRMs tissue resident macrophages rapidly disappear from the inflamed zone but slowly repopulate the organ by local proliferation; ii) monocyte-derived macrophages acquire multiple transcriptional phenotypes including one resembling tissue resident macrophages (despite the absence of TIMD4 and LYVE1 expression) and other transcriptional phenotypes possibly associated to tissue repair functions; iii) TIMD4^+^LYVE1^+^MHCII^low^ and CCR2^+^ TRMs pools present before the onset of acute injury orchestrate monocyte infiltration by controlling the diversity of inflammatory macrophages phenotypes with important consequences for the onset of tissue repair (Dick et al.). Further research is needed to establish a map of developmental trajectories linking monocyte subsets to macrophages recruited after myocardial injury, possibly involving a heterogenous spatial distribution (Dick et al.). Coillard and Segura detail and discuss the various markers and methodological options available, in human immunology settings, to probe the monocytic origin of tissue phagocytes (e.g., chimerism, labeling, and signature genes). However, the analysis of macrophage dynamics in the injured myocardium underlines the difficulties to move beyond the transcriptional definition of cellular populations to an integrated understanding of their functional contributions to both injury and tissue repair (Dick et al.).

Monocytes and macrophages play a major role in autoimmunity. In this topic, Ma et al. provide a broad panorama of the role of monocytes in multiple autoimmune conditions ranging from Systemic Lupus Erythematosus (SLE) to type 1 diabetes (T1D). Understanding monocyte biology has important therapeutic application. In this context, Hamilton delivers a synthesis of the current knowledge and controversies regarding the action of GM-CSF on monocytes and macrophages. A key feature of GM-CSF signaling lies in the activation of the CCL17 axis in monocyte-derived macrophages by a process involving the JMJD3 histone demethylase. Therapeutic blockade of GM-CSF in rheumatoid arthritis reduce CCL17 levels and reduces osteoarthritic pain (Hamilton).

## Monocyte Heterogeneity and the Regulation of Inflammation

The role of monocyte subsets in the resolution of inflammation is increasingly considered. The differentiation of classical monocytes into microbicidal macrophages is a central feature of anti-infectious innate immunity (7). However, this axis needs to be tightly regulated since microbicidal function can be associated to tissue damage. Postat and Bousso identify quorum sensing as new mechanism to maintain tissue integrity during the onset of anti-microbial innate immune reactions. In this case, nitric oxide release by differentiated anti-microbial monocytes plays a major role in activating the resolution of inflammation by a mechanism involving the suppression of mitochondrial respiration, thereby limiting cytokine and chemokine secretion (Postat and Bousso). Furthermore, IL-10 is also an important immuno-regulatory factor that can promote the acquisition of an immunoregulatory phenotype in differentiated moDCs associated to CD14 re-expression when combined with TLR stimulation (Krakow et al.). Undoubtedly, that understanding the mechanisms generating monocyte-derived cells endowed with immunosuppressive properties has a strong therapeutic relevance. For instance, Iglesias-Escudero et al. show in this topic that the presence of regulatory monocytes descendants is regulated by rapamycin in kidney transplant recipients.

The resolution of inflammation is a dynamic process relying on the efferocytic capacity of monocytes and their progeny, which support the development of anti-inflammatory responses and subsequently will contribute to the reestablishment of tissue resident macrophage pool. In this regard, Butenko et al. evidence that post-phagocytic “satiated” monocyte-derived macrophages express type I IFN and exhibit a transcriptomic profile limiting their pro-fibrogenic activity.

## Monocyte Heterogeneity and Cancer

Monocytes play a major role in cancer where CCR2-dependent classical inflammatory monocytes largely contribute to the pool of tumor-associated macrophages (TAMs), often with immunosuppressive and pro-tumoral properties (8). By contrast, non-classical monocytes had been shown to limit the metastatic spread of tumors through the blood circulation (9). Wu et al. discuss the relevance and limitations of the M1/M2 paradigm to understand the diversity of transcriptional states observed in TAMs. Wu et al. propose to re-assess TAM heterogeneity by integrating their ontogeny, activation and localization. Laviron and Boissonnas review the current knowledge on the selective contributions and functional contributions of acutely recruited monocytes in cancer versus pre-existing homeostatic tissue resident macrophage would they be derived from embryonic precursors or adult monocytes.

In summary, the very diverse and multidisciplinary contributions in this Frontiers TOPIC highlight the dynamism of monocyte research in finding new developments related to multiple biological processes and pathologies.

## Author Contributions

All authors listed have made a substantial, direct, and intellectual contribution to the work and approved it for publication.

## Conflict of Interest

The authors declare that the research was conducted in the absence of any commercial or financial relationships that could be construed as a potential conflict of interest.
